# DPV pUL15 possesses a potential NLS, which is important for the location of the terminase complex and for viral proliferation and genome cleavage

**DOI:** 10.1186/s13567-024-01420-9

**Published:** 2025-01-07

**Authors:** Qiao Yang, Yaya Feng, Lizhen Liu, Linlin Yang, Mingshu Wang, Ying Wu, Bin Tian, Xumin Ou, Renyong Jia, Dekang Zhu, Shun Chen, Mafeng Liu, Xinxin Zhao, Shaqiu Zhang, Juan Huang, Di Sun, Yu He, Zhen Wu, Ling Zhang, Yanling Yu, Anchun Cheng

**Affiliations:** 1 Engineering Research Center of Southwest Animal Disease Prevention and Control Technology, Ministry of Education, Chengdu, 611130 China; 2 Key Laboratory of Animal Disease and Human Health of Sichuan Province, Sichuan Agricultural University, Wenjiang, Chengdu City, 611130 Sichuan China; 3 Avian Disease Research Center, College of Veterinary Medicine, Sichuan Agricultural University, Wenjiang, Chengdu City, 611130 Sichuan China

**Keywords:** DPV, terminase, PUL15, NLS, genome cleavage

## Abstract

In herpesvirus, the terminase subunit pUL15 is involved in cleavage of the viral genome concatemers in the nucleus. Previous studies have shown that herpes simplex virus 1 (HSV-1) pUL15 can enter the nucleus without other viral proteins and help other terminase subunits enter the nucleus. However, this study revealed that duck plague virus (DPV) pUL15 cannot localize independently to the nucleus and can only be localized in the nucleus in the presence of pUL28 and pUL33. However, the data suggested the presence of a potential nuclear localization signal (NLS) in DPV pUL15, which is important for the localization of the terminase subunits. Subsequently, several single-point mutants were constructed to identify the vital amino acids within the NLS. The conserved amino acids K187, R188, and K190 are critical for the nuclear localization of pUL15, pUL28, and pUL33 but not for their interaction. Furthermore, corresponding recombinant viruses were constructed. The results revealed that the mutations rUL15K187Q, rUL15K188Q and rUL15K190Q had an obvious influence on concatemeric genome cleavage, but only K190Q significantly affected the production of progeny virions. These findings indicate that the NLS is important for the functions of DPV pUL15. Overall, a potential NLS and the key amino acids in DPV pUL15 were identified. Mutations in K187, K188 and K190 affected the cleavage of the concatemeric genome, but only mutations in K190 had an obvious effect on viral proliferation.

## Introduction

Duck plague virus (DPV), also called anatid herpesvirus, belongs to the Herpesviridae family, Alphaherpesvirinae subfamily, and Mardivirus genus, and its genome is similar in structure to that of other herpesvirus genomes [1–3]. The DPV genome consists of a unique long (UL) region, a unique short (US) region, and reverse repeats at both ends of the US, including an internal repeat sequence (IRS) and a terminal repeat sequence (TRS) [1, 4]. As observed for other herpesviruses, DPV genome replication results in large concatemers, which are cleaved into unit-length genomes during encapsidation. In general, herpesvirus genome encapsidation is mediated by the terminase complex, which is formed in the cytoplasm and subsequently enters the nucleus [5–7]. After specifically recognizing the *pac* sequence at the splice site of the concatemeric genome, the terminase complex performs a nuclease function to cleave the concatemeric genome, generating a packaging end [8–10]. The terminase then carries the concatemeric genome with the packaging end and binds to the portal protein on the capsid [11–13]. Moreover, the genome is translocated into the capsid through the energy generated by hydrolysing ATP [14–16]. When the genome is packaged into the capsid, the terminase exerts nuclease activity again and performs a second cleavage of the genomic DNA [17, 18]. The DNA package is then completed, and a mature C capsid containing the monomeric genome is formed. Finally, the terminase dissociates from the capsid and carries the remaining concatemeric genome for subsequent packaging rounds.

The herpesvirus terminase complex is believed to consist of three proteins: pUL15, pUL28, and pUL33 or their homologues [19–21]. In herpesvirus, pUL28 and its homologues recognize and bind to specific sequences in the viral genome, whereas pUL15 and its homologues hydrolyse ATP to provide energy for DNA translocation [22–24]. After completing the unit-length genome package, pUL15 may undergo conformational changes and exert nuclease activity to cleave the genomic DNA [22, 25]. Unlike those of pUL15 and pUL28, the exact functions of pUL33 and its homologues in herpesvirus are unclear, but pUL33 may play a regulatory role in the terminase complex [19, 26]. The absence of any terminase subunit blocks viral genome cleavage and packaging.

The components of the terminase complex and its role have been well studied in phages and some herpesviruses, but its role in DPV remains largely unknown. On the basis of the homology of the amino acid sequence, the DPV terminase complex is likely composed of pUL15, pUL28, and pUL33. In HSV-1, pUL15 contains a simian virus 40 (SV40) T-antigen-like NLS, 183-PKKRAKV-189, and can enter the nucleus in the absence of other viral proteins [27]. Moreover, HSV-1 pUL15 can carry pUL28 into the nucleus [28, 29]. Unlike HSV-1 pUL15, pUL89, the homologue of pUL15 in HCMV, lacks an NLS and cannot enter the nucleus [30]. Instead, pUL56, a homologue of pUL28, contains the NLS and can enter the nucleus without other viral proteins [31, 32]. Interestingly, another report indicated that pUL56, pUL89, and pUL51 in HCMV, which correspond to pUL28, pUL15, and pUL33 in HSV-1, respectively, cannot enter the nucleus independently but can enter the nucleus when the three proteins are coexpressed [30].

In DPV, DPV pUL15 does not possess the classic NLS and cannot enter the nucleus in the absence of other viral proteins [33]. Therefore, we explored the mechanism by which DPV pUL15 entered the nucleus. Additionally, we explored whether pUL15 has a potential NLS and its impact on viral genome cleavage.

## Materials and methods

### Cells and viruses

Duck embryo fibroblasts (DEFs), the human embryonic kidney (HEK) 293T cell line, and the chicken embryo fibroblast (DF-1) cell line were maintained at 37 °C and 5% CO_2_ in minimal essential medium (MEM, Gibco) supplemented with 10% foetal bovine serum (FBS, NEWZERUM). The DPV CHv strain and its mutants were cultured in DEF cells.

## Construction of plasmids

UL15, UL28, and UL33 gene fragments were obtained via PCR amplification from viral cDNA or DNA. EcoRI and BamHI were selected as the cloning sites. The UL15 gene was cloned and inserted into the pcDNA3.1 vector via homologous recombination and fused with three His tags at the C-terminus to generate the plasmid pcDNA3.1-UL15. The UL28 and UL33 genes were subsequently cloned and inserted into the pCMV vector between the EcoRI and XhoI restriction sites. The UL28 expression plasmid was fused with three HA tags at the N-terminus, whereas the UL33 expression plasmid was fused with three Myc tags at the N-terminus. All UL15 NLS mutants were generated via overlap PCR using the pcDNA3.1-UL15 plasmid as a template and cloned and inserted into pcDNA3.1. The GFP-SV40 NLS, GFP-UL15 NLS, 2×GFP, 2×GFP-SV40 NLS, 2×GFP-UL15 NLS, GFP-β-Gal, GFP-β-Gal-SV40 NLS and GFP-β-Gal-UL15 NLS plasmids were constructed on the basis of the pEGFP vector to detect the activity of the DPV pUL15 NLS. The plasmids encoding the GFP-UL15 NLS, 2×GFP-UL15 NLS and GFP-β-Gal-UL15 NLS contained amino acids 185–193 encoding the DPV pUL15 NLS. As positive controls, the GFP-SV40 NLS, 2×GFP-SV40 NLS and GFP-β-Gal-SV40 NLS plasmids contained amino acids 47–56 encoding the SV40 T NLS. Homologous recombination was performed with MonClone™ Hi-Fusion Cloning Mix V2 (Monad). The primers used for plasmid construction are listed in Table 1.

**Table 1:** **Primers for plasmid construction**

Primers	Primer sequence (5′- 3′)
pcDNA3.1-6His*3-UL15-F/R	F: CTTGGTACCGAGCTCGGATCCGAAATGGTAATGTTCGGGGCAACTT
	R: GTGCTGGATATCTGCACTAATGGTGATGGTGATGATGATGGTGATGGTGATGATGATGGTGATGGTGATGATGGA
pCMV-HA*3-UL28-F/R	F: TGGCCATGGAGGCCCGAATTCGGAAATACCCATACGATGTTCCAGATTACGCTTACCCATACGATGTTCCAGATTACGCTATGGAAAAAAATAGAGGTGC
	R: CCGCGGCCGCGGTACCTCGAGTTCATTCTGTCGAACTCCTTTCGAATTC
pCMV-Myc-UL33-F/R	F: TGGCCATGGAGGCCCGAATTCCCATGGACCGTGTAGAATC
	R: CCGCGGCCGCGGTACCTCGAGACTAACTATCGCGCAATATCTG
pcDNA3.1-F/R	F: AACTAGAGAACCCACTGCT
	R: CCCCAGAATAGAATGACACC
pCMV-HA/myc-F/R	F: GGTGGTGGTGCAAATCAAA
	R: AAGCAATAGCATCACAAATTTCAC
pcDNA3.1-His-UL15K185Q-F/R	F: TGGGACGGATGCAAATACTATACAAGCAAAGAGAAGT
	R: CATAAGTTGGCACATCGACCTTACTTCTCTTTGCTTGTATAGT
pcDNA3.1-His-UL15K187Q-F/R	F: TGGGACGGATGCAAATACTATAAAGGCACAAAGAAGT
	R: CATAAGTTGGCACATCGACCTTACTTCTTTGTGCCTTTATAGT
pcDNA3.1-His-UL15R188Q-F/R	F: TGGGACGGATGCAAATACTATAAAGGCAAAGCAAAGT
	R: CATAAGTTGGCACATCGACCTTACTTTGCTTTGCCTTTATAGT
pcDNA3.1-His-UL15K190Q-F/R	F: ACTATAAAGGCAAAGAGAAGTCAAGTC
	R: CATAAGTTGGCACATCGACTTGACTTCT
pcDNA3.1-His-UL15△NLS-F/R	F: GCAAATACTGGAAAGATGCGCGGTACTCTTG
	R: CTTTCCAGTATTTGCATCCGTCCCAGCATC
GFP-SV40 NLS-F/R	F: TTAGTGAACCGTCAGATCCGCTAGCGCTACCGGTCGCCACCATGGT
	R: CGAAGCTTGAGCTCGAGATCTGACCTTACGCTTCTTCTTTGGGAGTCCGGCCGGACTTGTACA
GFP-β-Gal-SV40 NLS-F/R	F: TTAGTGAACCGTCAGATCCGCTAGCGCTACCGGTCGCCACCATGGT
	R: CGAAGCTTGAGCTCGAGATCTGACCTTACGCTTCTTCTTTGGGAGTCCGGCCGGACTTGTACA
GFP-UL15 NLS-F/R	F: TTAGTGAACCGTCAGATCCGCTAGCGCTACCGGTCGCCACCATGGT
	R: CGAAGCTTGAGCTCGAGATCTATAAGTTGGCACATCGACCTTACTTCTCTTTGCCTTTATGAGTCCGGCCGGACTTGTACA
GFP-β-Gal-UL15 NLS-F/R	F: TTAGTGAACCGTCAGATCCGCTAGCGCTACCGGTCGCCACCATGGT
	R: CGAAGCTTGAGCTCGAGATCTATAAGTTGGCACATCGACCTTACTTCTCTTTGCCTTTATGAGTCCGGCCGGACTTGTACA
2×GFP -F/R	F: AGCTTCGAATTCTGCAGTCGACGGTACCGCATGGTGAGCAAGGGC
	R: GTTATCTAGATCCGGTGGATCCCGGGTTACTTGTACAGCTCGTC

### Construction of mutated infectious clones

On the basis of the bacterial artificial chromosome (BAC) rescue platform of DPV CHv strains previously established in our laboratory, a two-step labelled red recombination system was utilized to eliminate or mutate the UL15 NLS, resulting in the recombinant infection clones rUL15K187Q, rUL15R188Q and rUL15K190Q. Mutagenesis of these BAC clones was conducted according to a previously described protocol [34], and the mutagenesis primers used are listed in Table 2. In brief, the plasmid pEP-Kana-S served as a template, and the primers listed in Table 2 were used to amplify the targeted fragment containing Kana via PCR. The targeted fragment was then transferred into GS1783-ΔminiF bacteria containing the BAC-CHv genome. The Kana gene was subsequently removed, and Kana was identified via PCR using identification primers. Finally, the BAC clone containing the mutation site was confirmed by Sanger sequencing. The positive BAC clone was transfected into DEF cells for viral rescue, followed by serial passaging to obtain the recombinant virus. The repair BAC clones were prepared by reversing the added mutations.

**Table 2:** **Primers for infectious clone construction**

Primers	Primer sequence (5′- 3′)
pUL15K187Q-Kan-F/R	F: TTTCCAAGATGCTGGGACGGATGCAAATACTATAAAGGCACAAAGAAGTAAGGTCGATGTGCCTAGGGATAACAGGGTAATCGATTT
	R: CGCGCATCTTTCCATAAGTTGGCACATCGACCTTACTTCTTTGTGCCTTTATAGTATTTGCATGCCAGTGTTACAACCAAT
pUL15R188Q-Kan-F/R	F: CCAAGATGCTGGGACGGATGCAAATACTATAAAGGCAAAGCAAAGTAAGGTCGATGTGCCAACTAGGGATAACAGGGTAATCGATTT
	R: TACCGCGCATCTTTCCATAAGTTGGCACATCGACCTTACTTTGCTTTGCCTTTATAGTATTTGGCCAGTGTTACAACCAAT
pUL15K190Q-Kan-F/R	F: TGCTGGGACGGATGCAAATACTATAAAGGCAAAGAGAAGTCAAGTCGATGTGCCAACTTATGGTAGGGATAACAGGGTAATCGATTT
	R: CAAGAGTACCGCGCATCTTTCCATAAGTTGGCACATCGACTTGACTTCTCTTTGCCTTTATAGGCCAGTGTTACAACCAAT
UL15NLS-F/R	GAGGAAGAAACGATAGCACAT
	ATGTCTCAGCGATGTTTCAGA

### Coimmunoprecipitation and western blot analysis

HEK293T cells were plated on 6-well plates and transfected with pcDNA3.1-UL15, pCMV-UL28, pCMV-UL33 or pUL15 mutants using Lipofectamine™ 3000 Transfection Reagent (Invitrogen). Whole-cell lysates (input) were collected at 24 h post-transfection and centrifuged at 12 000 rpm for 10 min. The samples were incubated with mouse anti-His at 4 °C for 8 h, followed by the addition of protein G magnetic beads (Bio-Rad) at a ratio of 1:100 for 3 h. The protein-bound beads were washed with 0.2% Tris-buffered saline with Tween-20 (TBST) for 10 min, which was repeated 3 times, subjected to 10% SDS‒PAGE and transferred to a PVDF membrane. After being blocked with 5% BSA in TBST, the membrane was incubated with the indicated primary antibodies overnight at 4 °C. After being washed with TBST, the membrane was incubated with horseradish peroxidase (HRP)-conjugated goat anti-mouse IgG. Finally, a chemiluminescence assay was performed to detect the specific protein bands.

### Immunofluorescence assay

DF-1 cells were seeded on coverslips and transfected with the indicated plasmids. At 24 h post-transfection, the culture medium was removed, and the cells were fixed in paraformaldehyde and permeated with Triton X-100. Next, the cells were blocked with 3% BSA for 2 h and incubated with primary antibodies against mouse anti-His-Tag MAb (Proteintech), mouse anti-Myc-Tag MAb (Proteintech), rabbit anti-Myc-Tag pAb (Proteintech) or rabbit anti-HA-tag pAb (MBL) at 4 °C overnight. The cells were washed with PBST 3 times and co-incubated with the secondary antibodies TRITC-conjugated goat anti-rabbit IgG (Invitrogen) or FITC-conjugated goat anti-mouse IgG (Invitrogen). Finally, the cells were washed with PBST, and the nuclei were stained with DAPI diluted 1:1000. The samples were analysed with a fluorescence microscope.

### Growth curve

Monolayers of DEF cells in 12-well plates were infected with wild-type, mutant, or repair strains at a multiplicity of infection (MOI) of 1. At the indicated time points post-infection, the infected cells were harvested and lysed by freeze‒thawing 3 times to release the virus into the medium, after which the viral titre was determined by TCID_50_.

### TaqMan dual real-time PCR

TaqMan dual real-time PCR was performed according to a previously described method [1]. Briefly, DEF cells were seeded in 12-well plates and infected with wild-type or mutant strains at an MOI of 1, and whole-cell samples were harvested at 18 h post-infection. Viral DNA was extracted from harvested cells through phenol‒chloroform extraction. The total viral DNA copy number and viral concatemer DNA copy number were quantified via TaqMan dual real-time PCR using probes and primers for UL31 and the concatemeric terminal junction (CTJ) fragment (Tables 3 , 4).

**Table 3:** **Primers for TaqMan dual real-time PCR**

Primers	Primer sequence (5′- 3′)
Qpcr-CTJ-F/R	F: GGTGGAGTTGGCATGTTG R: CTTCCATAGCAGTGCATTGA
Qpcr-UL31-F/R	F: CCATGAGAGCCAGATCTTC R: CTCCCGTACTATGGCTAAC

**Table 4:** **Probes used in this study**

Probes	Probes sequence (5′- 3′)	5′-tag	3′-tag
QCTJ-P	CAAGCCACGCCCCTTTTGGC	VIC	MGB
QUL31-P	CGTACCGTACTGGCGACCGT	FAM	MGB

## Results

### DPV pUL15 displays different localizations in infected and transfected cells

**Figure 1:**
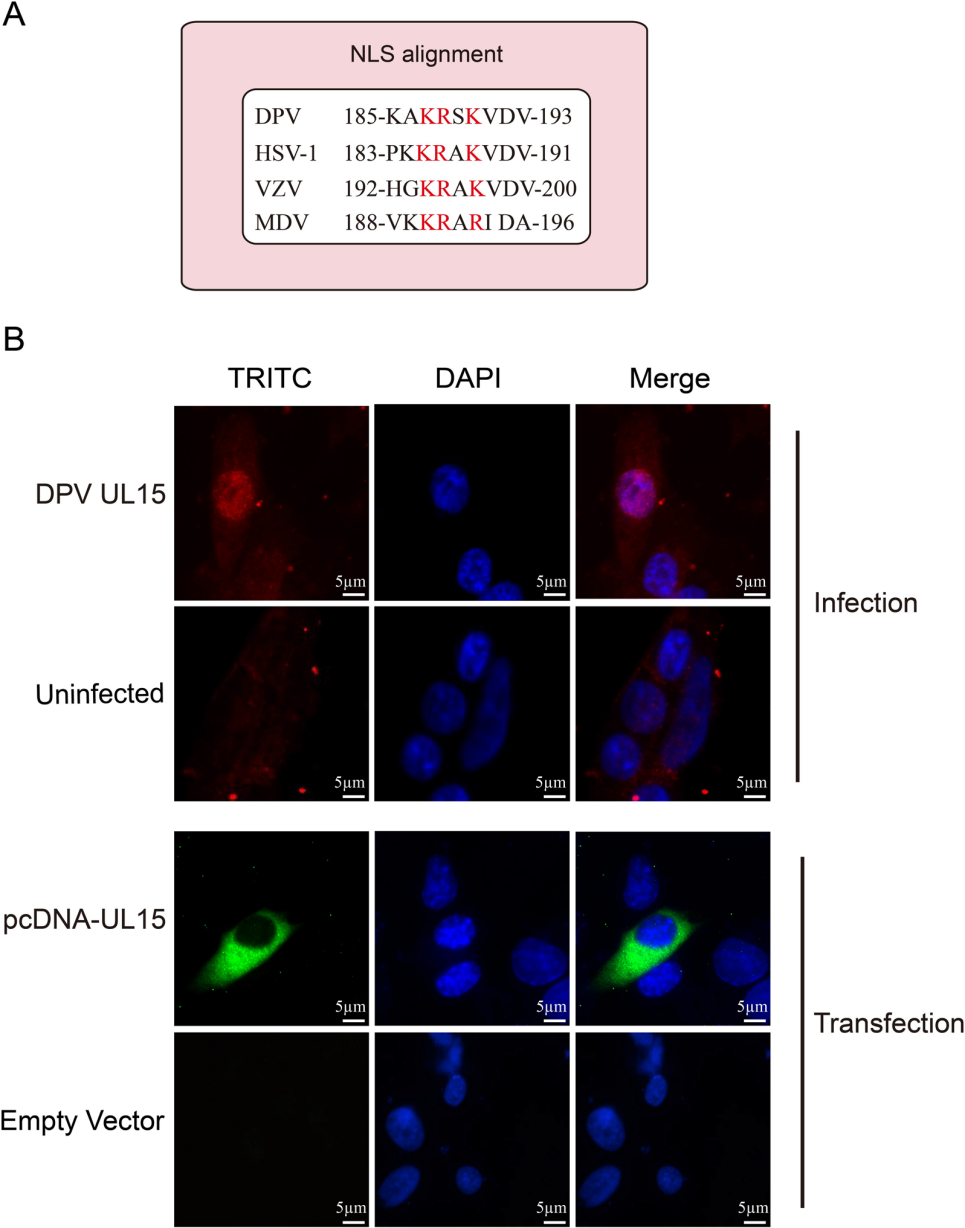
**Intracellular distribution of DPV pUL15 in infected and transfected cells. A** Alignment of the putative NLS sequence of DPV UL15 and its homologous protein. The accession numbers of DPV pUL15, HSV-1 pUL15, VZV ORF43 and MDV pUL15 are AFC61866.1, YP_009137089.1, NP_040165.1 and AEV55080.1, respectively **B** Localization of pUL15 in DEFs infected with DPV at 24 h post-infection (upper panel) and in DF-1 cells transfected with pcDNA3.1-pUL15 at 24 h post-transfection (lower panel). A mouse anti-pUL15 polyclonal antibody was used to detect pUL15 in DEFs infected with DPV, and a mouse anti-His tag antibody was used to detect pcDNA3.1-UL15.

DPV pUL15, a homologue of HSV-1 pUL15, is anticipated to localize primarily to the nucleus of infected cells. Sequence alignment also revealed that DPV pUL15 contained a putative NLS similar to that of HSV-1 pUL15 and that the key amino acids were conserved in the different homologues (Figure 1A). Our results revealed that DPV pUL15 displayed predominant nuclear localization in DEFs at 24 h post-infection (Figure 1B). A recombinant eukaryotic plasmid was constructed to detect the localization of pUL15 when it was expressed alone. However, DPV pUL15 was distributed mainly in the transfected cytoplasm of DF-1 cells in the absence of other viral proteins (Figure 1B).

### DPV pUL15 and other terminase subunits only enter the nucleus when co-expressed

**Figure 2:**
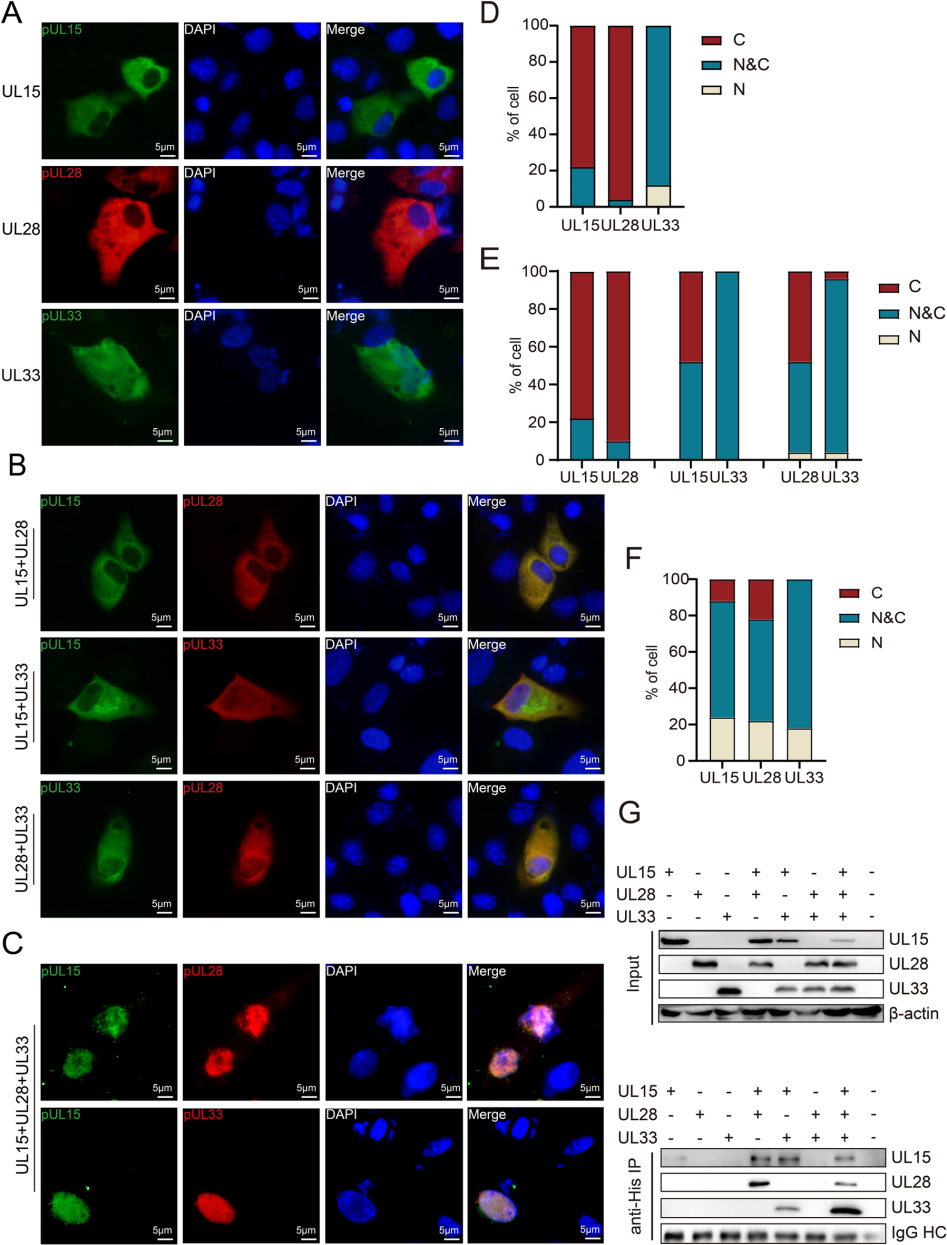
**pUL15 and other terminase subunits can only enter the nucleus when they are co-expressed and interact with each other. A** pUL15, pUL28 and pUL33 were transiently expressed in DF-1 cells. **B**,** C** pUL15, pUL28 and pUL33 were co-expressed in DF-1 cells as indicated. **D**–**F** The percentages of cells with cytoplasmic staining, nuclear staining, or nuclear and cytoplasmic staining corresponding to Figure A-C were determined by counting 50 fluorescent cells per group. N indicates localization only in the nucleus; N&C represents localization throughout the cell; C indicates localization only in the cytoplasm. **G** The interaction between terminase subunits was detected by Co-IP. pUL15, pUL28, and pUL33 were expressed or co-expressed in HEK293T cells, and the samples were harvested at 24 h post-transfection. β-actin and IgG HC (IgG heavy chain) served as loading controls.

In herpesvirus, pUL15, pUL28, and pUL33 assemble into a complex before entering the nucleus; thus, DPV pUL15 is expected to enter the nucleus in the presence of the other two terminase complex subunits. As a result, eukaryotic expression plasmids for pUL28 and pUL33 were constructed, and plasmids expressing pUL15, pUL28, and pUL33 were transfected into DF-1 cells separately or together. As shown in Figures 2A and D, when the three proteins were expressed alone, 96% of the pUL28 proteins and 78% of the pUL15 proteins were located in the cytoplasm. However, 88% of pUL33 exhibited a diffuse nucleocytoplasmic distribution when expressed alone, possibly due to its relatively small molecular weight (15 kDa). Notably, there was an increase in the number of cells where pUL15 (52%) or pUL28 (48%) were located in the karyoplasm when pUL15 or pUL28 was co-expressed with pUL33, respectively (Figures 2B and E). Finally, we co-expressed the three proteins and found that 24% of pUL15, 22% of pUL28, and 18% of pUL33 proteins were distributed in the nucleus, where they exhibited extensive colocalization (Figures 2C and F). These findings confirmed that the co-expression of terminase subunits could promote the enrichment of other subunits in the nucleus. These results suggested that the terminase subunits could interact with each other; thus, the three plasmids were co-transfected into HEK293T cells, and coimmunoprecipitation was used to detect the interactions between the three subunits (Figure 2G). The results showed that pUL15 interacted directly with pUL28 and pUL33 and that these three proteins could form a complex. In conclusion, the DPV terminase subunits pUL15, pUL28, and pUL33 can localize to the nucleus when co-expressed and interact with each other to form a complex.

### pUL15 NLS slightly enhances the nuclear localization of the fusion protein

**Figure 3:**
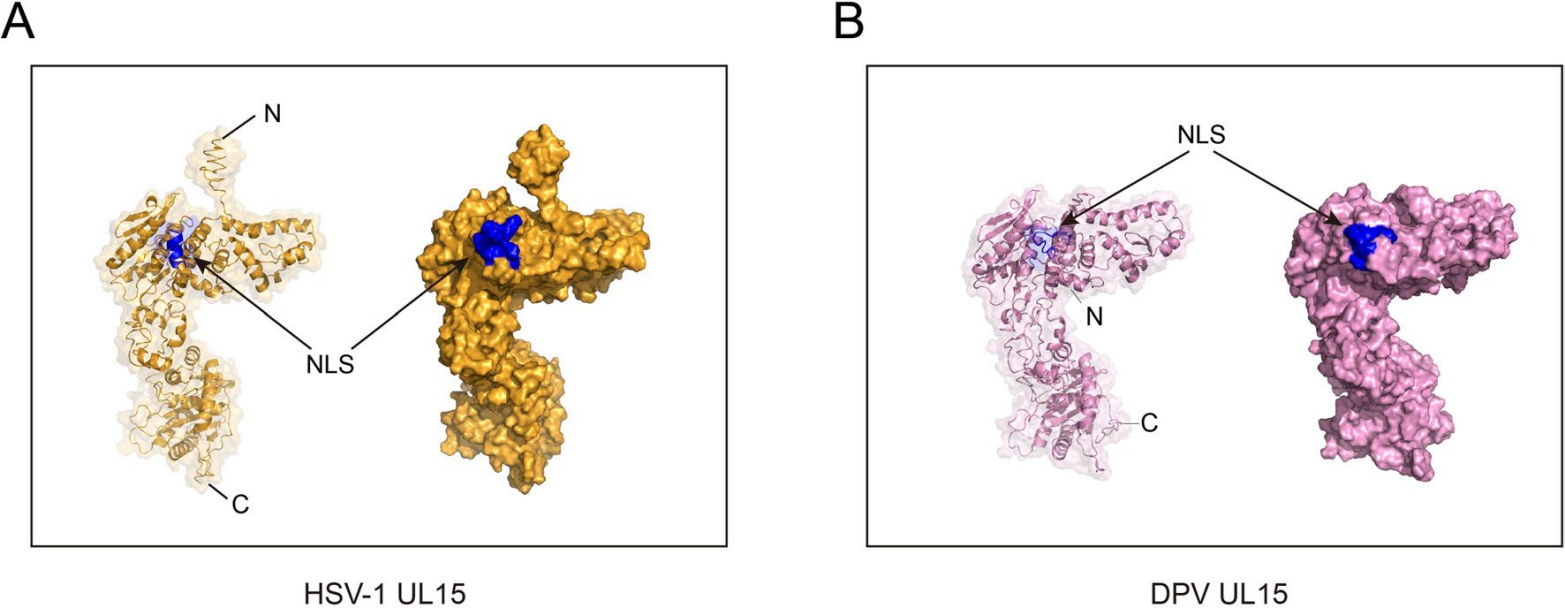
**Structural analysis of HSV-1 pUL15 and DPV pUL15. A** The protein structure of HSV-1 pUL15 was obtained from the Protein Data Bank, and the accession code is 6M5R. **B** The structure of DPV pUL15 was predicted by I-TASSER. The NLS is coloured in blue.

**Figure 4:**
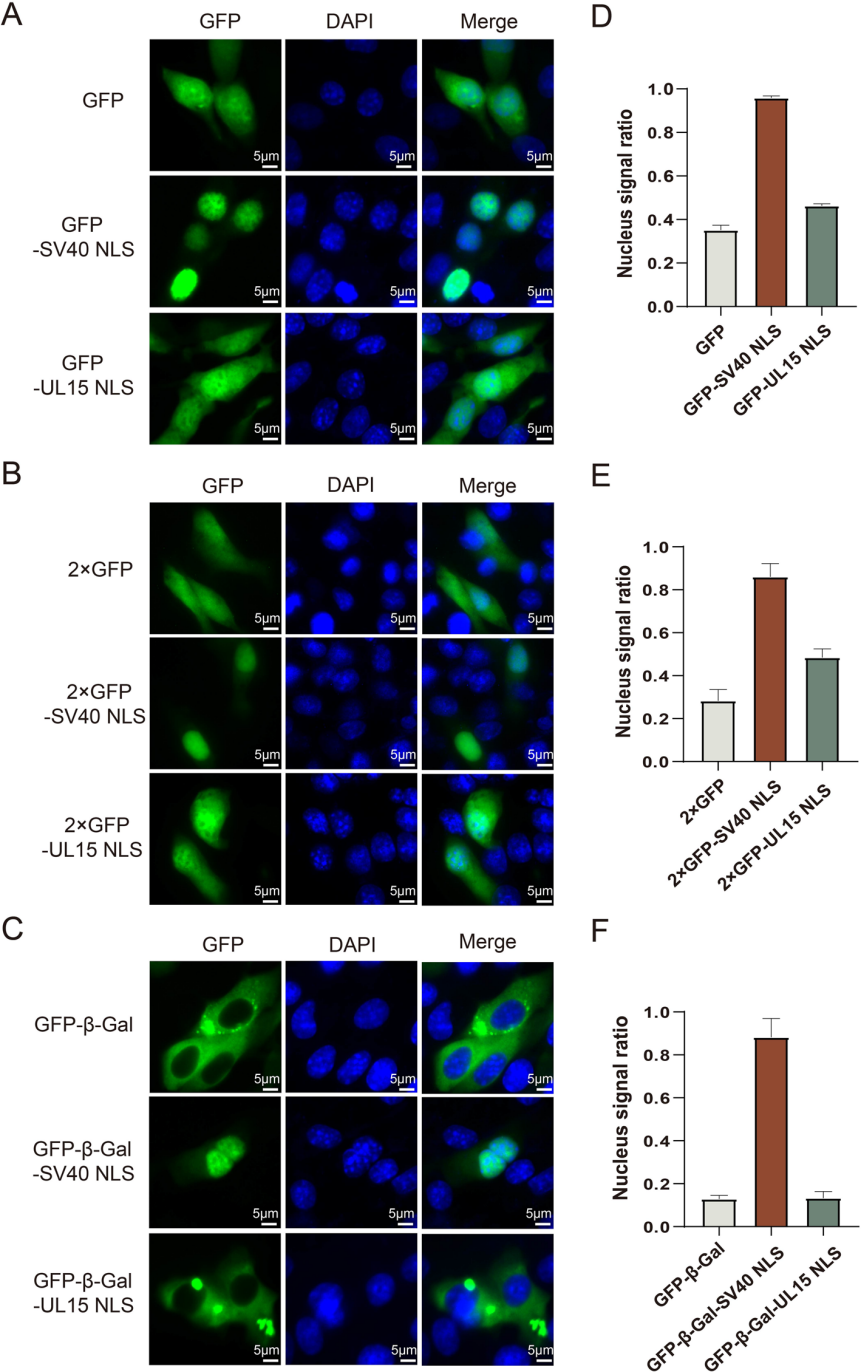
**The DPV pUL15 NLS enhanced the nuclear localization of the fusion protein. A** DPV pUL15 NLS was fused with GFP to detect the activity of the NLS. pEGFP was used as an empty vector control, and the SV40-T NLS fused to GFP was used as a positive control. The distribution of the fusion proteins was detected by observing green fluorescent protein. The fluorescence signal was analysed with ImageJ. **B** Each fusion protein was combined with another GFP, as shown in Figure 4A. The 2×GFP NLS, 2×GFP-SV40 NLS, and 2×GFP-UL15 NLS were expressed in DF-1 cells. **C** β-Gal was added to each fusion protein, as shown in Figure 4A. GFP-β-Gal, the GFP-β-Gal-SV40 NLS, and the GFP-β-Gal-UL15 NLS were expressed in DF-1 cells. **D**–**F** ImageJ software was used to analyse the fluorescence signal in each group and calculate the ratio of the fluorescence signal in the nucleus to that in whole cells. More than 30 cells were evaluated in each group.

According to the NLS prediction results, there was no NLS for DPV pUL15, pUL28 or pUL33. Therefore, it was speculated that amino acids 185–193 of pUL15 still represented an NLS due to the conservation of the amino acid sequence and structure of pUL15 (Figures 3A and B). To assess the activity of the pUL15 NLS, we generated various fusion constructs of the NLS to GFP, 2×GFP, and GFP-β-gal with molecular weights of 27 kDa, 54 kDa, and 116 kDa, respectively. The NLS of SV40-T served as a positive control and can carry foreign proteins into the nucleus. As shown in Figure 4, the negative controls, GFP and 2×GFP without an NLS, were located in both the nucleus and the cytoplasm (Figures 4A and B), and GFP-β-gal was distributed only in the cytoplasm (Figure 4C). The fluorescent signals of GFP or 2×GFP fused with the pUL15 NLS in the nucleus were stronger than the negative signals. The nuclear signal ratio of the GFP-UL15 NLS and 2×GFP-UL15 NLS was greater than that of the negative control, but this difference did not exceed 20% (Figures 4A, B, D, E). The GFP-β-gal-UL15 NLS was localized only in the cytoplasm, which was consistent with the finding that GFP-β-gal was present (Figure 4C and F). This result was thought to be due to the insufficient power of the DPV pUL15 NLS to help large proteins, such as beta gal, enter the nucleus. As a positive control, each fluorescent protein fused with the SV40 NLS was localized in the nucleus (Figures 4C and F). These results indicated that the NLS of DPV pUL15 could slightly enhance the ability of the foreign protein to enter the nucleus, although its ability was obviously weaker than that of the SV40 T NLS.

### pUL15 NLS is important for the nuclear localization of the terminase complex, and K187, R188 and K190 are key amino acids

**Figure 5:**
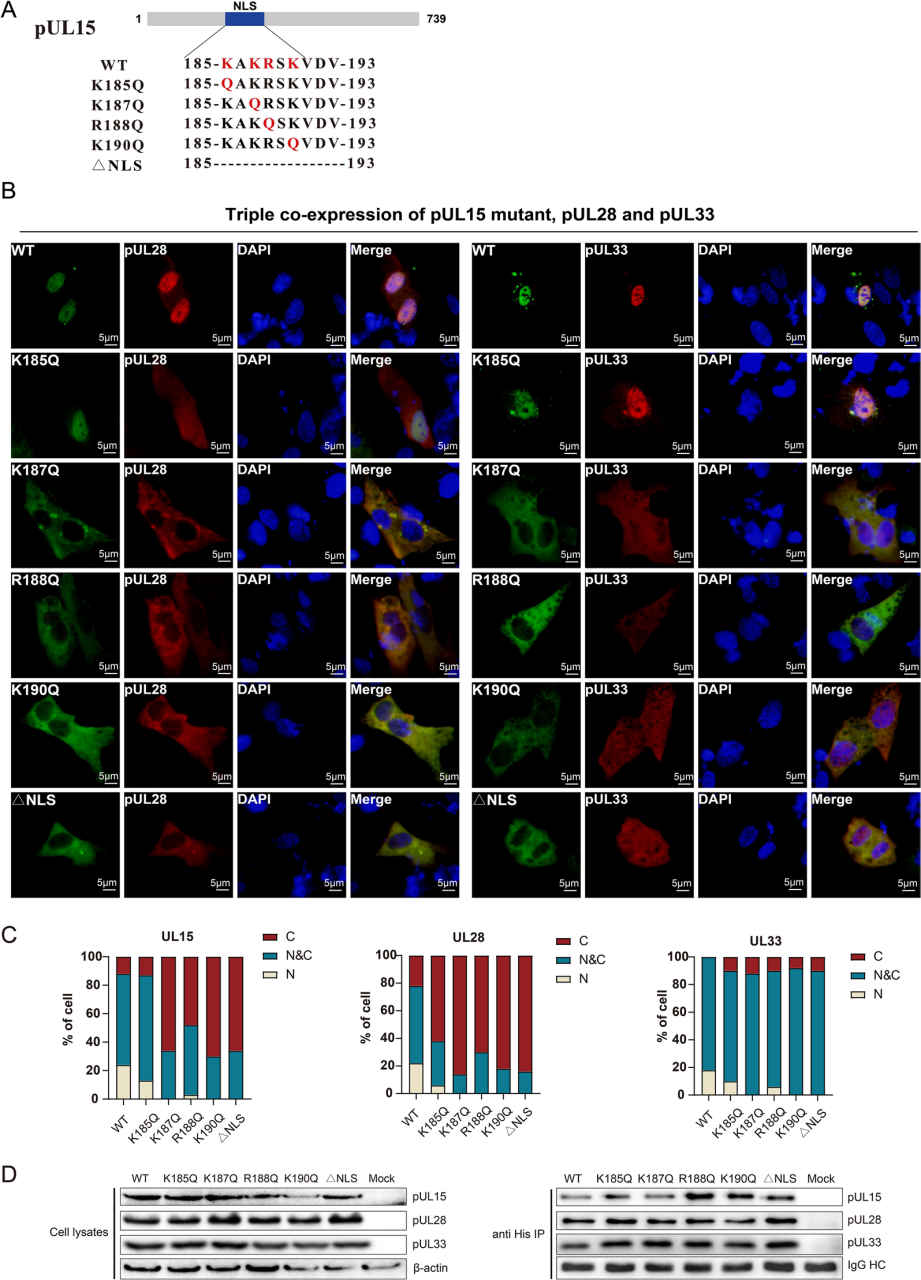
**Mutation of the DPV pUL15 NLS impaired the nuclear translocation of terminase subunits but not the interaction between terminase subunits. A** Schematic of DPV pUL15 NLS mutants. The basic amino acids (K or R) were replaced with glutamine (Q). **(B)** Mutations in pUL15 prevented the nuclear transportation of terminase subunits. pcDNA3.1-UL15K185Q, pcDNA3.1-UL15K187Q, pcDNA3.1-UL15R188Q, pcDNA3.1-UL15K190Q and pcDNA3.1-UL15∆NLS were co-transfected with pCMV-UL28 and pCMV-UL33 into DF-1 cells. Anti-His, anti-HA, and anti-Myc antibodies were used to detect the distribution of the pUL15 mutant, pUL28, and pUL33, respectively. **C** Statistical analysis of the different intracellular distributions of the terminase subunits shown in Figure 5B. The percentages of cells with only cytoplasmic staining, only nuclear staining, or nuclear and cytoplasmic staining corresponding to Figure 5B were determined by counting 50 fluorescent cells per group. **D** The interaction between pUL15 mutants, pUL28 and pUL33. The pUL15 mutants pUL28 and pUL33 were co-expressed in HEK293T cells as indicated. The interaction was detected by co-IP and Western blot analyses.

To further explore whether the NLS of pUL15 would affect the localization of the terminase subunits when co-expressed, a DPV pUL15 mutant lacking the NLS was constructed (Figure 5A). The NLS mutant plasmid of pUL15 was co-transfected into DF-1 cells with the expression plasmids pUL28 and pUL33. The location of each protein was detected by immunofluorescence, and the number of fluorescent cells was analysed (Figures 5B and C). The results revealed that deletion of the pUL15 NLS caused more than 60% of the proteins to localize in the cytoplasm, with no more than 40% of the pUL15 being distributed throughout the cell. pUL28 and pUL33 were also localized in the cytoplasm or throughout the cell. Generally, the basic amino acids in the NLS play a vital role in the nuclear import of proteins. There are four basic amino acids in the DPV pUL15 NLS: K185, K187, R188, and K190; therefore, we explored the critical amino acids. The four amino acids were replaced by glutamine (Q) because glutamine is a polar uncharged amino acid, and single point mutants, K185Q, K187Q, R188Q, and K190Q, were obtained (Figure 5A). Mutation at K185 had a minimal effect on terminase subunit localization, and the mutated NLS retained the ability to enter the nucleus, indicating that the lysine at 185 was not necessary for the nuclear localization of terminase subunits. Notably, the K187Q, R188Q, and K190Q mutants significantly altered the subcellular localization of pUL15, pUL28 and pUL33 (Figures 5B and C). These results revealed a significant reduction in the nuclear localization of K187Q, R188Q, and K190Q and the co-expression of pUL28 and pUL33. It was difficult to identify the protein localized in the nucleus when K187, K188, and K190 were mutated, which indicated that although the pUL15 NLS was not strong enough, it still played a critical role in transporting the terminase subunits into the nucleus. The basic amino acids, especially K187, R188, and K190, are important for the function of the DPV pUL15 NLS.

### DPV pUL15 NLS is dispensable for the formation of the terminase complex

To determine whether the change in the localization of the terminase subunits was simply due to the loss of NLS function rather than an effect on subunit interactions, various mutants of pUL15 were co-expressed with pUL28 and pUL33 in 293T cells, after which whole-cell lysates were analysed via a coimmunoprecipitation (co-IP) assay (Figure 5D). Anti-His tag antibodies successfully precipitated pUL28 and pUL33 from lysates of the three protein-co-expressing 293T cells prepared at 24 h post-transfection. Although the basic amino acids in the DPV pUL15 NLS were mutated or deleted, pUL15 could still form complexes with pUL28 and pUL33. This result further demonstrated that the altered localization of each terminase subunit was attributed to the inactivation of the DPV pUL15 NLS and not to the disruption of subunit interactions.

### The pUL15 NLS is important for viral proliferation and cleavage of the viral concatemeric genome

**Figure 6:**
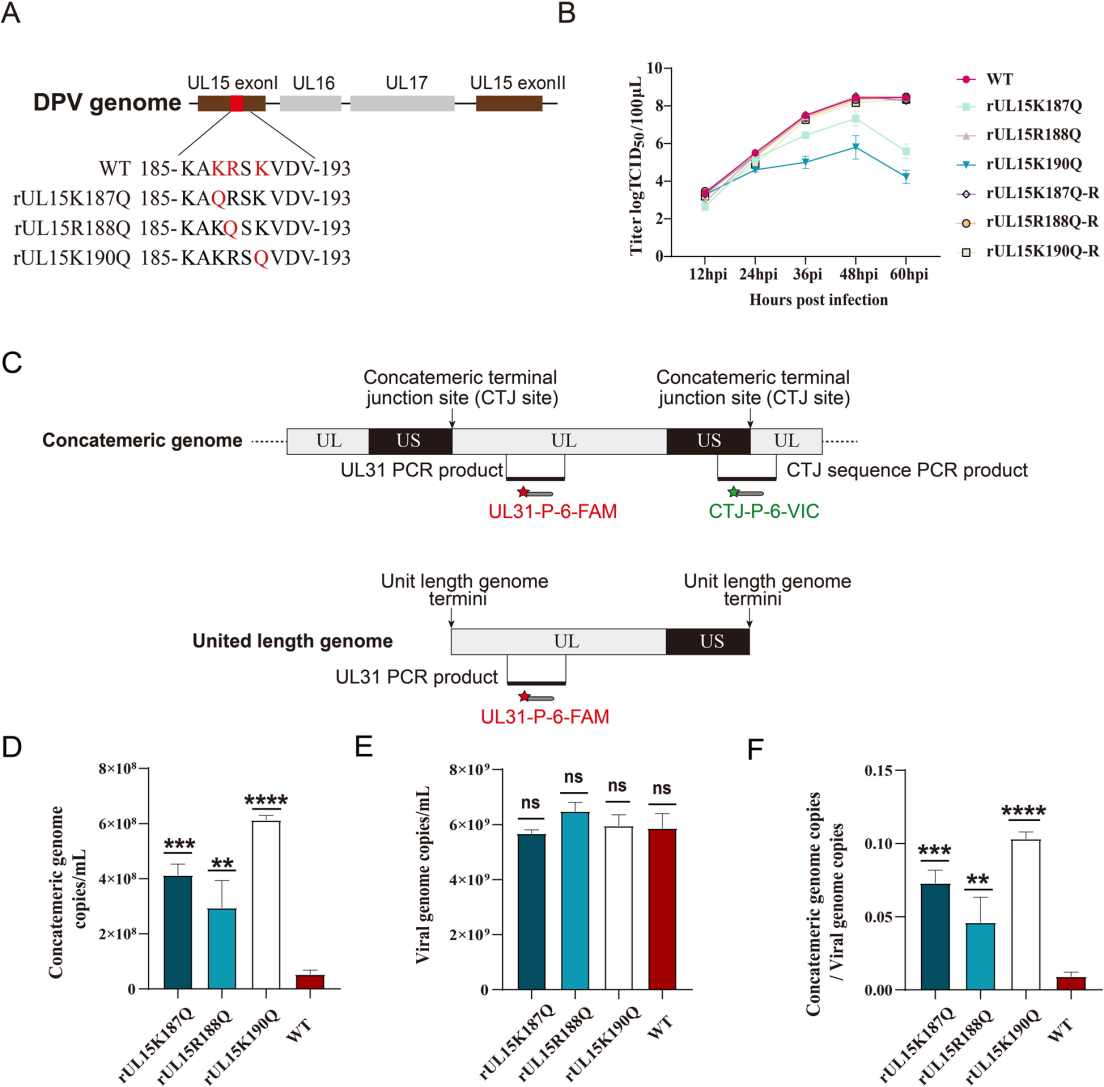
**DPV pUL15 K187**,** R188 and K190 are important for viral proliferation and genome cleavage. A** Schematic diagram of recombinant infectious clone construction. **B** Growth curves of DPV pUL15 mutants. **C** Schematic of TaqMan dual real-time PCR. The UL31-P-6-FAM probe was used to detect the number of total genome copies, and the CTJ-P-6-VIC probe was used to detect the number of concatemeric genome copies. **D** Number of concatemeric genome copies of WT, rUL15K187Q, rUL15R188Q, and rUL15K190Q at 18 h post-infection. **E** The total number of genome copies of WT, rUL15K187Q, rUL15R188Q, and rUL15K190Q was detected at 18 h post-infection. **F** The ratio of concatemeric genome copy number to total number of genome copies.

To analyse the influence of pUL15 NLS mutants on the process of viral replication, the recombinant mutant strains rUL15K187Q, rUL15R188Q, and rUL15K190Q and the corresponding repair strains were constructed (Figure 6A). The results revealed that the titres of rUL15K187Q and rUL15K190Q were lower than those of the WT and repair strains and that the mutation in K190 had the most significant effect on the production of progeny viruses (Figure 6B).

The herpesvirus genome is replicated into the concatemeric genome, which needs to be cleaved by terminase in the nucleus. The correct localization of terminase is therefore critical for concatemer cleavage during infection. In this study, TaqMan dual real-time PCR was used to detect cleavage of the concatemeric genome. The copy number of the UL31 gene represented the copy number of the total viral DNA, and the copy number of the CTJ sequence represented the copy number of the concatemeric DNA (Figure 6C). There was no significant difference in the number of total viral DNA copies between the mutant and WT strains in the infected cells, indicating that the viral genome could still replicate even when the NLS of pUL15 was mutated (Figure 6E). However, the number of concatemeric DNAs in all the mutants was greater than that in the WT (Figure 6D), suggesting that the mutations in the DPV pUL15 NLS inhibited concatemer cleavage, resulting in concatemer accumulation. An analysis of the ratio of the concatemeric genome copy number to the total viral genome copy number of the mutants revealed that it was significantly greater than that of the WT (Figure 6F).

## Discussion

The herpesvirus concatemeric genome is cleaved by the terminase complex, and a single genome copy is translocated into the capsid. Previous studies have shown that the herpesvirus terminase complex localizes to the nucleus during the later stages of infection, but not all terminase subunits can independently enter the nucleus. HSV-1 pUL15 can independently enter and help other terminase subunits enter the nucleus [35]. However, pUL15 of DPV cannot enter the nucleus on its own, even though it can enter the nucleus in infected cells [33]. Interestingly, when DPV pUL15 was co-expressed with pUL28 and pUL33, all three proteins entered the nucleus. This result differed from that of HSV-1 but was similar to that of HCMV terminase homologues, even though DPV and HCMV belong to different herpesvirus subfamilies [30, 35–37]. Given these findings, it was hypothesized that DPV pUL15 might not have an NLS and that other terminase subunits with an NLS might carry pUL15 into the nucleus. However, NLS predictions revealed that neither pUL28 nor pUL33 possessed an NLS. On the basis of these results, we reconsidered the possibility that the conserved amino acids 185–193 in DPV pUL15 served as an NLS. Typically, a strong NLS, such as the SV40 NLS, can carry GFP or GFP-β-gal into the nucleus [38–40]. In this case, the nuclear signal ratio of the GFP-UL15 NLS and 2×GFP-UL15 NLS increased slightly, and the fused protein could not completely enter the nucleus. This result was in agreement with our expectations because DPV pUL15 could not enter the nucleus in the absence of other viral proteins. It was speculated that the DPV pUL15 NLS was functional only when pUL15 formed the entire complex with pUL28 and pUL33 or that DPV pUL15 together with other terminase subunits could comprise a complete, novel nuclear localization signal to help each subunit enter the nucleus.

To further confirm the NLS activity of DPV pUL15 and identify key amino acids, the NLS was deleted, or a single amino acid was mutated. A conventional NLS comprises a cluster of basic amino acids that are recognized by cellular transport proteins [41, 42]. The DPV pUL15 NLS contains four basic amino acids, which are positively polar charged amino acids. In the DPV pUL15 NLS, the basic amino acids are replaced with polar uncharged amino acids to conserve the global polarity of the protein. The results revealed that deletion of the NLS was sufficient to prevent the nuclear localization of pUL15 and the other two terminase subunits, strongly suggesting that 185–193 is important for the nuclear localization of DPV pUL15 and is responsible for transporting the terminase complex into the nucleus. The DPV pUL15 NLS is composed of a cluster of four basic amino acids: K185, K187, R188, and K190. K187 and R188 correspond to K185 and R186 in HSV pUL15, the two conserved amino acids in the NLS of pUL15 homologues in the α subfamily [27, 42]. Sequence alignment indicated that K190 was the third conserved basic amino acid, and it was replaced with another key basic amino acid, arginine, in Marek’s disease virus (MDV). K185 was not conserved and had no corresponding amino acid in other pUL15 homologues. Site-directed mutagenesis of single amino acids, K187, R188, and K190, was sufficient to prevent efficient nuclear localization. The only exception, K185, which slightly affected the localization of terminase subunits, was perhaps because it was not a critical amino acid for the NLS function of DPV pUL15. Moreover, co-IP experiments excluded the possibility that mutation or deletion of the DPV pUL15 NLS resulted in the inability to form the terminase complex. The above results suggest that amino acids 185–193 of DPV pUL15 represent a potential NLS and are important for the localization of the terminase complex in the nucleus even though the NLS cannot serve as a classic NLS, such as the SV40 NLS. Amino acids K187, R188, and K190 are involved in the transport of terminase subunits into the nucleus.

**Figure 7:**
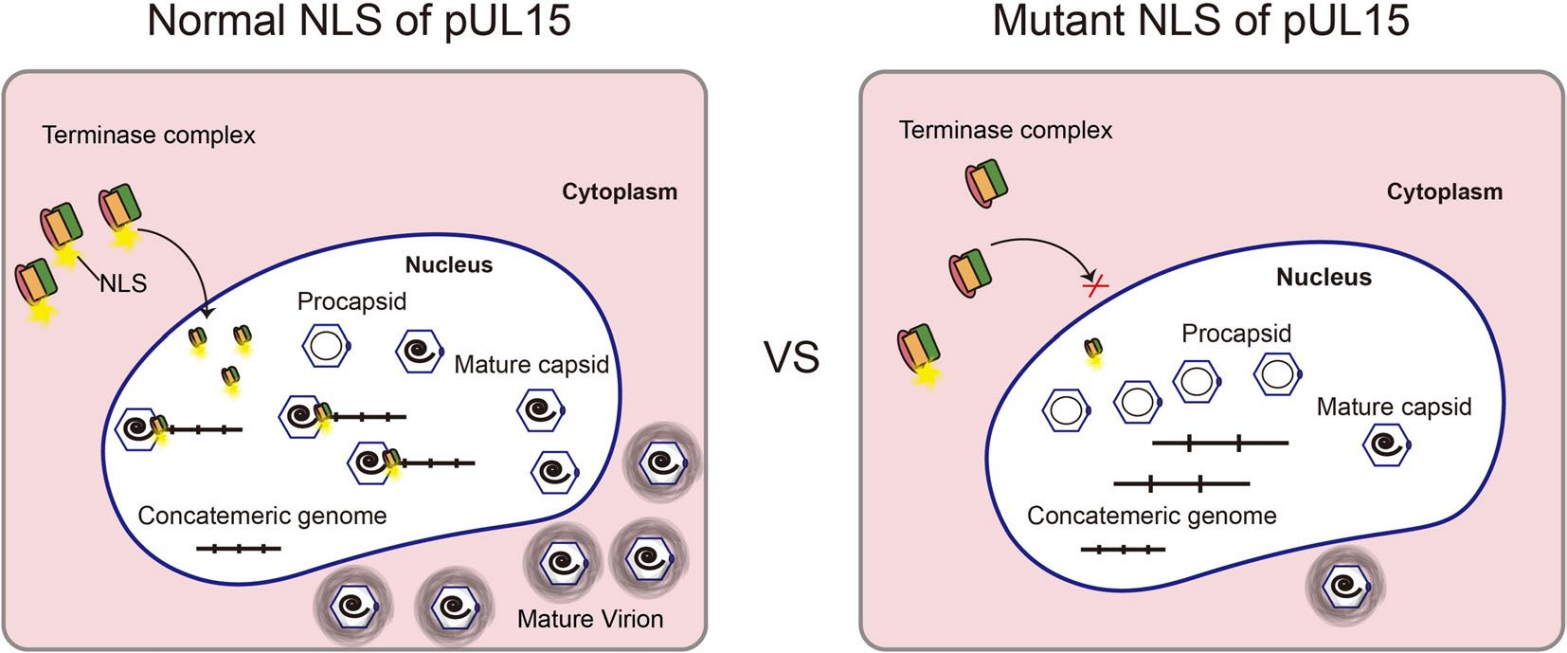
**Schematic diagram of the role of the DPV pUL15 NLS in terminase complex nuclear transport and viral genome cleavage.**

It has been reported that HSV-1 UL15-null and point mutation viruses can produce viral DNA but cannot carry out cleavage [43, 44]. This study revealed that three NLS mutants, K187Q, R188Q, and K190Q, inhibited cleavage of the concatemeric genome but not DNA synthesis. Compared with those of the wild-type virus, the titres of the K187Q and K190Q mutants decreased 100–200-fold. These findings suggested that mutations in K187 and K190 impaired viral proliferation. However, the titre of the R188Q mutant was similar to that of the wild-type virus, which was different from its effect on genome cleavage. Considering that the function of K188Q was compensated by other amino acids during viral proliferation, there was no obvious difference in titre between the mutant K188Q and wild-type viruses. These results indicated that inactivation of the DPV pUL15 NLS affected the localization of terminase subunits and the functions of terminase. It is believed that inactivation of the DPV pUL15 NLS prevents the terminase complex from entering the nucleus, thereby affecting the cleavage of the viral genome and the production of progeny virions (Figure 7).

In summary, we demonstrated that DPV pUL15 can enter the nucleus only in the presence of two other terminase subunits, pUL28 and pUL33. The three subunits, pUL15, pUL28, and pUL33, interact and form a complex. Although the NLS of DPV pUL15 cannot serve as a classic NLS, it is still important for the nuclear import of terminase subunits, and K187, R188, and K190 are the key amino acids for potential NLS function. Mutations in K187, R188, and K190 also impaired the cleavage of viral concatemers, but only the K187Q and K190Q mutants, especially the K190Q mutant, influenced viral proliferation.

## Data Availability

The datasets used and/or analysed during the current study are available from the corresponding author upon reasonable request.
